# Genome-wide search for candidate genes for yeast robustness improvement against formic acid reveals novel susceptibility (Trk1 and positive regulators) and resistance (Haa1-regulon) determinants

**DOI:** 10.1186/s13068-017-0781-5

**Published:** 2017-04-19

**Authors:** Sílvia F. Henriques, Nuno P. Mira, Isabel Sá-Correia

**Affiliations:** Institute for Bioengineering and Biosciences, Department of Bioengineering, Instituto Superior Técnico, Universidade de Lisboa, Av. Rovisco Pais, 1049-001 Lisbon, Portugal

**Keywords:** Formic acid tolerance, Formic acid toxicity, Chemogenomic analysis, Trk1, Haa1, Lignocellulosic hydrolysates, Yeast robustness

## Abstract

**Background:**

Formic acid is an inhibitory compound present in lignocellulosic hydrolysates. Understanding the complex molecular mechanisms underlying *Saccharomyces cerevisiae* tolerance to this weak acid at the system level is instrumental to guide synthetic pathway engineering for robustness improvement of industrial strains envisaging their use in lignocellulosic biorefineries.

**Results:**

This study was performed to identify, at a genome-wide scale, genes whose expression confers protection or susceptibility to formic acid, based on the screening of a haploid deletion mutant collection to search for these phenotypes in the presence of 60, 70 and 80 mM of this acid, at pH 4.5. This chemogenomic analysis allowed the identification of 172 determinants of tolerance and 41 determinants of susceptibility to formic acid. Clustering of genes required for maximal tolerance to this weak acid, based on their biological function, indicates an enrichment of those involved in intracellular trafficking and protein synthesis, cell wall and cytoskeleton organization, carbohydrate metabolism, lipid, amino acid and vitamin metabolism, response to stress, chromatin remodelling, transcription and internal pH homeostasis. Among these genes is *HAA1* encoding the main transcriptional regulator of yeast transcriptome reprograming in response to acetic acid and genes of the Haa1-regulon; all demonstrated determinants of acetic acid tolerance. Among the genes that when deleted lead to increased tolerance to formic acid, *TRK1*, encoding the high-affinity potassium transporter and a determinant of resistance to acetic acid, was surprisingly found. Consistently, genes encoding positive regulators of Trk1 activity were also identified as formic acid susceptibility determinants, while a negative regulator confers protection. At a saturating K^+^ concentration of 20 mM, the deletion mutant t*rk1Δ* was found to exhibit a much higher tolerance compared with the parental strain. Given that t*rk1Δ* accumulates lower levels of radiolabelled formic acid, compared to the parental strain, it is hypothesized that Trk1 facilitates formic acid uptake into the yeast cell.

**Conclusions:**

The list of genes resulting from this study shows a few marked differences from the list of genes conferring protection to acetic acid and provides potentially valuable information to guide improvement programmes for the development of more robust strains against formic acid.

**Electronic supplementary material:**

The online version of this article (doi:10.1186/s13068-017-0781-5) contains supplementary material, which is available to authorized users.

## Background

Formic acid (p*K*_a_ = 3.74; octanol–water partition coefficient, LogP = −0.54 [1]) is the simplest monocarboxylic acid in nature. The worldwide production of this acid is in large part (35%) used to prevent fungal and bacterial growth in silage [2]. Formic acid is also among the inhibitors found in lignocellulosic hydrolysates, typically at concentrations around 30 mM although they are variable depending on the type of treatment used to prepare the hydrolysates [3–6]. A comprehensive understanding of the mechanisms underlying tolerance to formic acid is therefore important to guide the design of more robust industrial yeast strains able to more efficiently use lignocellulosic hydrolysates as growth medium in biorefineries [7].

The straightforward exploitation of Omic approaches combined with metabolic engineering and synthetic biology strategies in *Saccharomyces cerevisiae* is considered instrumental to guide the improvement of yeast strains for production of biofuels and added-value chemicals [8–14]. The application of Omic analyses to elucidate the genes and the pathways involved in yeast adaptive response and tolerance to stress induced by weak acids, in particular acetic acid, has been essential to identify their molecular targets and guide the design of more robust strains for efficient production of organic acids or to cope with their presence in the lignocellulosic hydrolysates and to their increased concentration during fermentation [8, 11, 15–19]. However, equivalent studies to unravel the molecular determinants of toxicity and tolerance to formic acid, especially at a genome-wide scale, are scarce [20].

As described for other weak acids, it is believed that the undissociated form of formic acid may enter the yeast cells through simple diffusion and dissociate in the cytosol, leading to the disruption of the electrochemical gradient across the plasma membrane, to the decrease of internal pH and the triggering the production of reactive oxygen species (ROS) [20–22]. Adaptation to weak acids involves the increase of activity of plasma membrane H^+^-ATPase and vacuolar H^+^-ATPase to counteract the acidification of cytosolic pH and the dissipation of plasma membrane H^+^ gradient [23, 24]. Several other mechanisms of toxicity and tolerance to weak acids have been reported [8, 9, 15, 16, 18]. Recently, genes involved in lipid metabolism emerged as determinants of yeast response and tolerance to acetic acid stress [15], the Tor complex 2-Ypk1 signalling pathway being involved in this response [25].

Key regulatory pathways involved in yeast global response and tolerance to weak acids have been unveiled exploring genome-wide approaches [15, 16, 18, 26]. Among them is the signalling pathway regulated by the transcription factor Haa1. Yeast response to short-chain weak acids, as it is the case of acetic, propionic and lactic acids [18, 27], is regulated by Haa1, which is considered the main orchestrator of the reprograming of yeast transcriptome in response to acetic acid [28]. Indeed, Haa1 regulates a large regulon including 80% of the acetic acid-stress responsive genes, including those involved in transcription (e.g., *MSN4*, *MCM1*, *FKH2* and *COM2*), multidrug resistance (MDR) transport (e.g., *TPO2*, *TPO3* and *AQR1*), cell wall remodelling (e.g., *YGP1* and *SPI1*), nucleic acid processing (e.g., *SAP30*) and in lipid (e.g., *YCP1* and *SUR2*) and carbohydrate metabolism (*HRK1*) [15, 28]. Considering the central role of Haa1 in yeast adaptive response and tolerance to acetic acid and other low-chain fatty acids, the manipulation of the Haa1-signalling pathway either by increasing the expression of *HAA1*, based on the expression of extra copies of the gene and/or the use of more efficient promoters [29–33], or by the insertion of beneficial mutations in the coding sequence [33, 34], was successfully attempted.

Formic acid was found to be co-utilized with glucose by *S. cerevisiae* in glucose-limited cultures (7.5 g L^−1^) under aerobic conditions to generate NADH molecules for respiratory dissimilation [35, 36]. This weak acid is rapidly converted into CO_2_ and water by formate dehydrogenases (FDH) encoded by *FDH1* and *FDH2* genes with NADH molecules being produced in the process, and this activity was proposed to contribute to formic acid detoxification [36–38]. In mammalian and plant cells, formate is believed to bind cytochrome c oxidase (complex VI of the electron transport chain), which catalyses the reduction of molecular oxygen to water using cytochrome c as the electron donor [39, 40]. The inhibition of this last step of the electron transport chain hampers proton gradient maintenance at physiological values and ultimately ATP synthesis [41]. In mammalian cells, this process was found to be accompanied by an increase of ROS production (i.e., superoxide anions and hydroxyl radicals) in the mitochondria, leading to the oxidative damage of proteins, lipids and DNA [42–45]. With the exception of a proteomic analysis [20], little has been done to understand the cytotoxic effects of formic acid and the response mechanisms in *S. cerevisiae*. This study is a first attempt to identify, in a comprehensive manner, genes involved in yeast tolerance and susceptibility to formic acid at a genome-wide scale. Among the selected determinants identified through the chemogenomic analysis performed is the Haa1 regulon, for the first time demonstrated to be required for tolerance to formic acid. Quite unexpectedly, the high-affinity potassium transporter Trk1 [46] and the positive regulators of its activity emerged as susceptibility determinants to this acid. Trk1 activity is positively regulated by the Serine/threonine protein kinases Sat4 and Hal5 [47], and negatively regulated by the Serine/threonine protein phosphatase Ppz1 [48, 49]. The activity of the Trk1 transporter is also positively regulated by the activity of Hal3, a negative regulatory subunit of the protein phosphatase Ppz1 [48]. Contrasting with the results from this study, *TRK1* was found to confer protection against acetic acid in yeast [15]. Moreover, yeast susceptibility to acetic acid was found to be attenuated by growth medium supplementation with K^+^ ions [15]. Considering the results obtained in this study, the unexpected role of Trk1 in *S. cerevisiae* tolerance to formic acid was examined.

## Methods

### Strains and growth media

The haploid parental strain *S. cerevisiae* BY4741 (*MATa*, *his3Δ1*, *leu2Δ0*, *met15Δ0*, *ura3Δ0*) and the derived collection of single deletion mutants, in which each non-essential gene was individually deleted, were obtained from EUROSCARF (Frankfurt, Germany). Yeast cells were batch cultured in YPD medium containing 2% (w/v) glucose (Merck), 2% (w/v) yeast extract (VWR International) and 1% (w/v) peptone (VWR International), or in MM4 medium containing 1.7 g L^−1^ Yeast Nitrogen Base (YNB) w/o amino acids or ammonium (Difco), 20 g L^−1^ glucose, 2.65 g L^−1^ (NH_4_)_2_SO_4_ (Scharlau), supplemented with 20 mg L^−1^ methionine, 30 mg L^−1^ lysine, 60 mg L^−1^ leucine, 40 mg L^−1^ tryptophan, 20 mg L^−1^ histidine and 20 mg L^−1^ uracil (all from Sigma-Aldrich). Cell cultures were cultivated at 30 °C with orbital agitation (250 rev min^−1^). To test the growth of wild-type BY4741 and *trk1Δ*- or *trk2Δ*-derived strains under potassium limitation, an ammonium phosphate-derived medium was used, containing 0.492 g L^−1^ MgSO_4_·7H_2_O (Merck), 0.02 g L^−1^ CaCl_2_ (Sigma-Aldrich), 1.056 g L^−1^ (NH_4_)_2_HPO_4_ (Merck), 3.96 g L^−1^ (NH_4_)_2_SO_4_, 20 g L^−1^ glucose (Merck), 2 mg L^−1^ niacin, 2 mg L^−1^ pyridoxine, 2 mg L^−1^ thiamine, 2 mg L^−1^ pantothenate, 0.02 mg L^−1^ biotin, 20 mg L^−1^ methionine, 60 mg L^−1^ leucine, 20 mg L^−1^ histidine and 20 mg L^−1^ uracil supplemented with the desired concentration of KCl (all from Sigma). A 3 M formic acid stock solution (Sigma-Aldrich) was prepared in water and the pH of the solution adjusted to 4.0 or to 4.5 with NaOH depending on the growth medium supplementation. Solid media were prepared by addition of 20 g L^−1^ agar (IberAgar) to the liquid media.

### High-throughput screening of the deletion mutant collection

To screen the EUROSCARF deletion mutant collection for susceptibility or tolerance to formic acid, strains were grown for 16 h in MM4 medium in 96-well plates as described before [15, 16]. Using a 96-pin replica platter, the cell suspensions were spotted onto the surface of MM4 solid medium acidified with HCl to pH 4.5 and supplemented, or not, with formic acid to a final concentration of 60, 70 or 80 mM. Depending on the severity of growth inhibition, plates were incubated at 30 °C for 2 or 3 days. Two datasets containing genes identified as determinants of resistance (Additional file 1, Fig. 1a) or of susceptibility (Additional file 2, Fig. 1b) to formic acid were obtained. Both datasets were clustered according to biological process Gene Ontology (GO) assignments using the MIPS functional catalogue (http://mips.helmholtz-muenchen.de/funcatDB; over-representation of functional categories was considered for a *p* value ≤0.01) and this analysis was complemented using the information available in *Saccharomyces* Genome Database (SGD) (http://www.yeastgenome.org). The simultaneous occurrence of genes from the same dataset in a shared metabolic pathway or signalling pathway was determined using the Kyoto Encyclopedia for Genes and Genomes (KEGG) Mapper Search pathway tool (http://www.genome.jp/kegg/tool/map_pathway1.html).

**Fig. 1:**
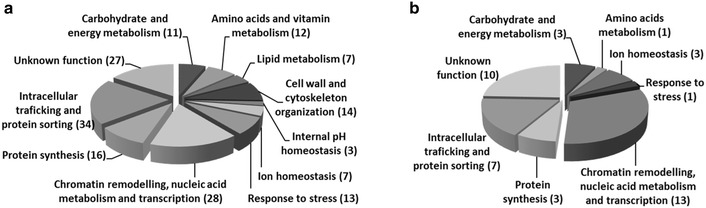
Biological functions found to be enriched in the datasets of genes found to confer tolerance or susceptibility to formic acid. **a** Genes found to confer tolerance to formic acid, listed in Additional file 1, were clustered according their biological process GO assignments using the MIPS functional catalogue (http://mips.helmholtz-muenchen.de/proj/funcatDB), and functional categories were considered to be over-represented if *p* value ≤0.01. **b** Genes found to confer susceptibility to formic acid were manually clustered according to the information available in SGD (http://www.yeastgenome.org) into the indicated categories. The number of genes identified within each category is indicated in *brackets*

### Susceptibility assays to formic acid of selected strains

The susceptibility of selected *S. cerevisiae* BY4741-derived deletion mutants was compared with the parental strain in shake flasks or petri dishes in MM4 medium (pH 4.0) or in solid MM4 medium (pH 4.5). To test the strain susceptibility to formic acid (30 mM) in liquid MM4 medium, mid-exponential cells pre-grown in MM4 medium (pH 4.0) to an optical density at 600 nm (OD_600_) of 0.8 ± 0.05 were used to inoculate the same basal medium, either or not, supplemented with 30 mM formic acid, with an initial OD_600_ of 0.05 ± 0.005. Growth curves were followed based on the increase of the OD_600_ and by determining the number of colony-forming units (CFU) per ml of cell suspension in solid YPD medium. To test the susceptibility of selected mutants to formic acid in solid MM4 medium (pH 4.5), mid-exponential cells grown in MM4 liquid medium (pH 4.5) to a final OD_600_ of 0.8 ± 0.05 were diluted to an OD_600_ of 0.05 ± 0.005 and spotted onto solid medium supplemented or not with formic acid (50 and 80 mM). Plates were incubated for 3 days at 30 °C.

The susceptibility of wild-type BY4741 and *trk1Δ* and *trk2Δ* mutants to formic acid was also compared by spot assays performed in the presence of increasing concentrations of potassium. The solid ammonium phosphate-derived medium (at pH 4.5) was supplemented with KCl (stock solution of 2 M) to final concentrations of 0.5, 2 and 20 mM and supplemented or not with formic acid at the indicated concentrations. The cells used to inoculate the agar plates were grown until an OD_600_ of 0.4 in liquid ammonium phosphate-derived medium (at pH 4.5) supplemented with 20 mM of KCl. Cells were diluted in sterile H_2_O to an OD_600_ of 0.05, and this solution was used to prepare 1:3 and 1:15 diluted suspensions. Four microliters of each cell suspension were spotted onto solid media and the plates incubated at 30 °C for 5 days, depending on the severity of growth inhibition.

### Effect of formic acid in the transcriptional level from Haa1-regulated genes

Real time RT-PCR was used to compare mRNA levels from a number of Haa1-target genes (*TPO2*, *TPO3*, *COM2*, *HRK1*, *SAP30* and *SUR2*) in BY4741 and BY4741*haa1Δ* genetic backgrounds, cultivated in MM4 medium (pH 4.0) supplemented or not with 30 mM of formic acid, as described above for formic acid susceptibility assays, except that the initial OD_600_ of the growth curves which was set at 0.4 ± 0.05. Cells incubated for 30 min in presence or absence of 30 mM of formic acid were harvested by centrifugation at 5000*g*, 4 °C, for 3 min and the pellet immediately frozen in liquid nitrogen and stored at −80 °C until used. Total RNA was extracted from these pellets using the hot phenol method [50], treated with DNAaseI (Invitrogen) according to the manufacturer instructions, and 1 μg of the treated RNA was used in the reverse transcription step (Taqman^®^ reverse transcription reagents). Primers used for the PCR amplification of the selected genes (Additional file 3) were designed using the Primer Express Software (Applied Biosystems), and 62.5 ng of the synthesized cDNA were used as template for the amplification step. *ACT1* gene was used as the endogenous control. Values of mRNA determined for BY4741 cells cultivated in the absence of the acid were used as normalization factor, being the mRNA levels from the different genes determined for the same RNA sample set as 1.

### Cellular accumulation assays for [^14^C]formic acid

BY4741 and BY4741*trk1Δ* cells, pre-grown in MM4 medium (pH 4.0), were used to inoculate fresh medium with an initial OD of 0.1 and cultivated at 30 °C with orbital agitation (250 rev min^−1^) until an OD_600_ of 0.3–0.5 was reached. An appropriate volume of cell suspension was filtered and cells resuspended in 5 ml of fresh MM4 medium (pH 4.0) with an OD_600_ of 0.5 ± 0.05 and incubated for 10 min at 30 °C (150 rev min^−1^) before the accumulation experiment was performed. The experiment started after the addition of cold formic acid (final concentration of 30 mM) and 2.5 μL of [^14^C]formic acid ([^14^C]sodium formate, 1 mCi ml^−1^, ARC0163A, American Radiolabeled Chemicals). At the indicated time points, 200 μL of the cell suspension were filtered through glass microfiber filters (Whatman GF/C) and washed twice with ice-cold TM buffer containing 100 mM MES and 41 mM Tris buffers (both from Sigma, pH of 4.0 adjusted with HCl). The filters containing the adsorbed cells were placed into scintillation vials containing 5 ml of scintillation cocktail (Ultima Gold MV, PerkinElmer) and the radioactivity of the samples measured in a Beckman LS 5000TD scintillation counter. The radioactivity of 100 μL of the culture supernatant was also measured at each time point. The accumulation ratio of intracellular/extracellular [^14^C]formic acid at the indicated time points was calculated considering that the internal volume (*V*_i_) of cells of both strains was equal to 2.5 μL per mg of dry weight [23]. The difference between the means determined for wild-type and *trk1Δ* cells were considered statistically significant for *p* values <0.05 using a one-way ANOVA.

## Results

### Genome-wide identification of *S. cerevisiae* genes that confer tolerance to formic acid

The EUROSCARF collection, containing approximately 5000 single deletion mutant strains devoid of all non-essential *S. cerevisiae* genes, was screened to identify genes contributing to tolerance to formic acid-induced stress, based on the identification of those mutants exhibiting increased susceptibility to 60, 70 or 80 mM of formic acid at pH 4.5, when compared to the parental strain (Additional file 4). This chemogenomic analysis allowed the identification of a total of 172 tolerance genes (Additional file 1). Two levels of susceptibility were considered, depending on the severity of growth inhibition of the deletion mutants when compared to the wild-type strain, as illustrated in Additional file 4. All genes that were found to be determinants of tolerance to formic acid (despite the level of susceptibility conferred by the deletion of the gene) are indicated in Additional file 1. These genes were grouped using the MIPS functional catalogue (http://mips.helmholtz-muenchen.de/proj/funcatDB). The different functional categories identified as being over-represented (*p* value ≤0.01) in the dataset, when compared to the genome and the list of genes and the number of genes in each category, are depicted in Fig. 1a and in Additional file 1.

### Haa1 and the Haa1-regulon are involved in response and tolerance to formic acid

**Fig. 2:**
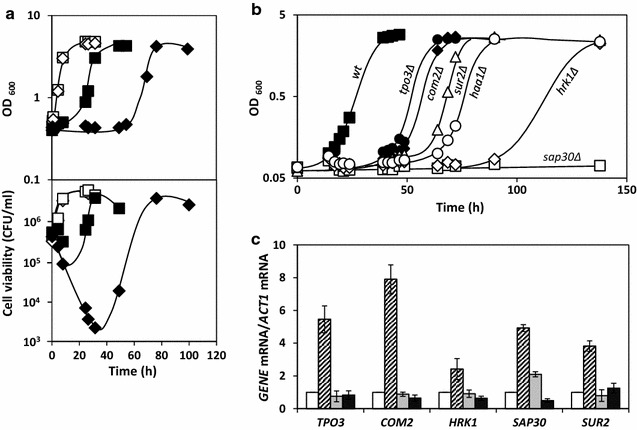
The expression of *HAA1* and of Haa1-regulated genes is required for *S. cerevisiae* adaptation and tolerance to formic acid. **a** Cells of the parental strain (*squares*) and deletion mutant *haa1Δ* (*diamons*) were grown in liquid MMB medium (pH 4.0) supplemented (*closed symbols*), or not (*open symbols*), with 30 mM of formic acid (*upper panel*) and growth was followed based on culture optical density at 600 nm (OD_600_). Cell viability (CFU ml^−1^) was also determined during cultivation (*lower panel*). **b** Growth curves of the parental BY4741 strain and of strains with the Haa1-target genes deleted were incubated in liquid MMB medium (pH 4.0) supplemented with 30 mM of formic acid. The *growth curves* indicated in* panels* (**a**) and (**b**) are representative of at least three independent experiments. **c** Comparison of the mRNA levels from *HAA1* and selected Haa1-regulated genes assessed in wild-type cells incubated in basal medium (*white bars*), or exposed to formic acid (30 mM) for 30 min (*diagonal stripes bars*) and in *haa1Δ* cells in cultivated in those same conditions (*grey* and *black bars*, respectively). The *bars* represent the average values of at least three independent experiments, and the *error bars* the associated standard deviation

Among the tolerance genes identified based on the deletion mutant collection screening, those encoding the transcription factor Haa1 and genes of the Haa1-regulon (direct or indirect target genes for Haa1 regulation under acetic acid stress) were selected for further studies. In fact, their prominent role in yeast adaptive response to acetic acid and other short-chain fatty acids has been proposed. To confirm the putative role of Haa1 and of genes of the Haa1-regulon in yeast response and tolerance to formic acid, the susceptibility to this weak acid of the parental strain and of several individual deletion mutants with genes of the Haa1-regulon deleted, in particular, *tpo2Δ*, *tpo3Δ*, *com2Δ*, *sur2Δ*, *hrk1Δ* and *sap30Δ*, was compared based on formic acid-induced increase of the duration of the latency period for these mutant strains when cultivated in MM4 medium (pH 4.0) supplemented with 30 mM of formic acid (Fig. 2b). With the exception of *TPO2* gene (data not shown), all the Haa1-regulated genes tested were shown to contribute significantly to yeast tolerance to formic acid, exerting a significant effect by decreasing the duration of formic acid-induced lag-phase (Fig. 2b). As described for acetic acid [15], a particularly marked effect of *HRK1* and *SAP30* deletion in the increase of formic acid susceptibility was observed, being even higher than the phenotype registered for cells with the *HAA1* deleted (Fig. 2b).

The transcriptional levels from the Haa1-target genes *TPO3*, *COM2*, *SUR2*, *HRK1 and SAP30* were also compared in parental strain and *haa1Δ* mutant during early response to formic acid (Fig. 2c) to test this weak acid effect in the hypothesized increase of mRNA levels from Haa1-target genes and its dependence on Haa1. To compare the mRNA levels from the above referred genes, the parental and *haa1Δ* cells were cultivated in MM4 medium at pH 4.0 and collected after 30 min of incubation in medium supplemented, or not, with 30 mM of formic acid (Fig. 2c). Transcription levels from selected Haa1-target genes under formic acid stress were found to be significantly higher compared with those in unstressed parental cells, while this formic acid-induced transcriptional activation of the target genes was drastically reduced in cells with the *HAA1* gene deleted, supporting the idea that Haa1 is an activator of these genes increased transcription in response to formic acid stress (Fig. 2c). The mRNA levels from the *SAP30* gene in unstressed *haa1Δ* cells were found to be higher than those registered in the parental strain but no likely justification can be put forward for this result at this time.

### Genome-wide identification of *S. cerevisiae* genes whose expression increases susceptibility to formic acid

The chemogenomic analysis carried out also led to the identification of 41 genes whose individual deletion results in increased tolerance to formic acid of each single mutant when compared to the parental strain, as illustrated in Additional file 4. According to the MIPS functional catalogue, no significant enrichment in GO terms (*p* value ≤0.01) could be identified, probably due to the high variety of functions associated to this small gene dataset. For this reason, the identified determinants of susceptibility to formic acid were manually grouped according to their description in SGD (www.yeastgenome.org) into the following categories: “carbohydrate and energy metabolism”, “amino acids metabolism”, “ion homeostasis”, “response to stress”, “protein synthesis”, “intracellular trafficking”, “protein sorting” and “chromatin remodelling, nucleic acid metabolism and transcription” (Additional file 2 and Fig. 1b). Among the genes found to contribute to increased yeast susceptibility to formic acid, two genes are highlighted: the gene *CYC3*, encoding a cytochrome c heme lyase, and the *ARN2* gene, encoding a transporter of siderophore–iron complexes involved in iron uptake.

**Fig. 3:**
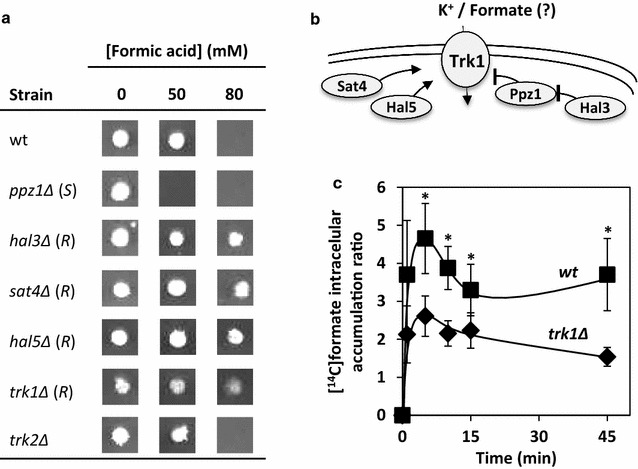
*TRK1* gene and genes encoding positive/negative regulators of Trk1 activity are determinants of susceptibility/tolerance to formic acid. **a** Wild-type (wt), *trk1Δ* and deletion mutants where the genes coding for regulators of Trk1 activity (*HAL3*, *SAT4*, *HAL5* and *PPZ1*) were deleted were tested for their susceptibility to formic acid in solid MMB medium (pH 4.5) supplemented with the acid at the indicated concentrations. Resistance (*R*) and susceptibility (*S*) phenotypes. **b** Schematic representation of the positive and negative regulators of Trk1 activity. **c** Time-course accumulation of [^14^C]formic acid in non-adapted wild-type cells and *trk1Δ* cells cultivated in MM4 growth medium (at pH 4.0) after exposure to 30 mM of formic acid during a 45-min period. Values represent the average of at least three independent experiments and the *error bars* represent the associated standard deviation. The *asterisks* indicate a significant different (*p* value <0.05) between the accumulation of [^14^C]formic acid in wild-type and *trk1Δ* cells at that time point

From the EUROSCARF collection screening, genes *SAT4* and *HAL5*, two positive regulators of Trk1 activity, were found to confer susceptibility to yeast. Based on this result, it was considered of interest to systematically compare the susceptibility to formic acid of an individual mutant with the *TRK1* gene deleted and of mutants with the positive (*HAL3*, *SAT4* or *HAL5)* and negative (*PPZ1)* regulators [47, 51] of its activity individually deleted (Fig. 3a, b). Surprisingly, results indicate that the *TRK1* gene, encoding the high-affinity potassium transporter [46], as well as genes *SAT4* and *HAL5* are determinants of susceptibility to formic acid (Additional file 2, Fig. 3a), suggesting a detrimental effect of the biological activity of Trk1 in yeast protection to formic acid. Consistent with this hypothesis, the mutant with the gene encoding the negative regulator of Trk1, Ppz1, deleted is more susceptible to formic acid than the parental strain, whereas the mutant deleted for the gene encoding the negative regulator of Ppz1, Hal3, is more tolerant to the acid (Fig. 3a, b).

**Fig. 4:**
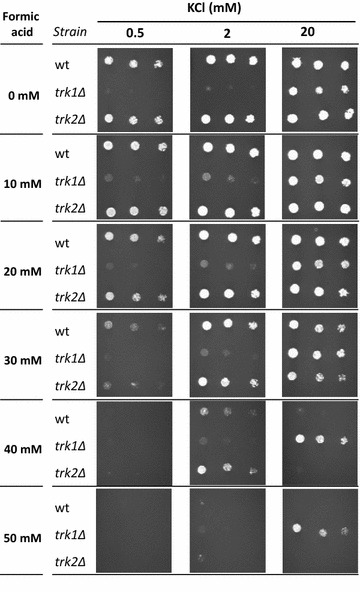
Effect of potassium concentration in tolerance to formic acid of yeast cells with different capacities of K^+^ uptake. Comparison of the susceptibility of wild-type (wt) BY4741, *trk1Δ* and *trk2Δ* deletion mutants to different concentrations of formic acid (0, 10, 20, 30, 40 and 50 mM) in ammonium phosphate medium supplemented with increasing concentrations of KCl (0.5, 2 and 20 mM). Serial dilutions (1:0, 1:3 and 1:15) of a water-diluted cellular suspension with an OD_600_ of 0.05 were spotted onto agar plates and incubated during 5 days at 30 °C

However, the deletion of *TRK2*, coding for the low-affinity potassium transporter, had no detectable effect on yeast susceptibility to formic acid (Fig. 3a). Since the screening of the deletion mutant phenotypes was performed at a non-limiting K^+^ concentration (8 mM), to understand the conjugated effect of potassium availability and the presence of Trk1 or Trk2 in yeast tolerance to formic acid, the susceptibility to formic acid of wild-type BY4147 cells and *TRK1* or *TRK2* deleted mutants was compared by spot assays at K^+^ concentrations ranging from 0.5 to 20 mM of KCl (Fig. 4). At the higher concentration of 20 mM of KCl, ectopic uptake of K^+^ is sufficient to fulfil cellular requirements [52]. Results show that the deletion of the *TRK1* gene increases, at the highest K^+^ concentration tested, the tolerance of yeast cells to high concentrations of formic acid, allowing growth at 40 and 50 mM under the experimental conditions used (Fig. 4). However, the effect of *TRK2* gene deletion was not detected at any of the K^+^ concentrations tested (Fig. 4). Based on these results, it was hypothesized that the high-affinity K^+^ transporter, Trk1, besides its main biological function, may also facilitate the uptake of formic acid/formate into the yeast cell. This hypothesis is consistent with the higher accumulation of radiolabeled [^14^C]formic acid in the parental strain compared with *trk1Δ* cells (Fig. 3c).

## Discussion

From the chemogenomic analysis performed in this study, several genes belonging to the functional categories “intracellular trafficking and protein sorting”, “chromatin remodelling, nucleic acid metabolism and transcription” and “intracellular pH homeostasis” were found to be over-represented in the dataset of the genes required for yeast adaptation and tolerance to formic acid. These categories were also found to be enriched among the genes required for yeast maximal tolerance to acetic and propionic acids [15, 16]. Vacuolar protein sorting-associated genes have been implicated in multidrug resistance (MDR) [53], and acetic acid-triggered intracellular acidification was shown to lead to trafficking defects, hampering vesicle shuffling from endosome to vacuole [54]. Since formic acid is believed to share part of the cytotoxic effects associated to acetic and propionic acids, in particular the induction of intracellular acidification, the protective effect conferred by the genes grouped in those categories against formic acid was not surprising.

Genes involved in oxidative stress response were also found to confer tolerance to formic acid, consistent with recent evidences indicating that formic acid induces yeast apoptosis, accompanied by the increase of ROS production [20]. Also, the expression of the *ARN2* gene, encoding a transporter for siderophore-iron complexes involved in iron uptake, was found to increase yeast susceptibility to formic acid possibly due to iron ability to catalyse ROS production via Fenton reaction. Collectively, these results indicate that formic acid induces oxidative stress and cell damage.

The deletion of *CYC3*, encoding the enzyme that catalyses the insertion of an heme group into the apo-form of cytochrome c [55], was found to confer susceptibility to formic acid. In yeast, *CYC3* deletion leads to increased H_2_O_2_ levels and consequently to increased oxidative stress [56]. However, our results suggest that the deletion of the *CYC3* gene is beneficial for yeast tolerance to formic acid. Since formate is believed to bind cytochrome c oxidase (complex VI of the electron transport chain), specifically inhibiting cytochrome c oxidase activity [39, 40, 45], this effect may affect the re-oxidation of cytochrome c with consequences for the performance of *CYC3*-expressing cells.

Two genes (*GPD2* and *GPP2*) involved in glycerol biosynthesis were also identified as determinants of tolerance to formic acid, as well as *FPS1*, encoding a aquaglyceroporin involved in the control of glycerol homeostasis [54, 57]. These results are in line with those reported for non-*S. cerevisiae* yeast strains exhibiting higher intracellular levels of glycerol, presumably acting in osmoprotection [58]. The production of glycerol by glycerol 3-phosphate dehydrogenase (*GPD2*) contributes to reduce the NADH pool which is important since formate dehydrogenase activity generates NADH and is believed to be required for formic acid detoxification [36]. For the same reason, the role of *GPP2*, involved in the catalysis of the last step of glycerol synthesis, is also a determinant of formic acid tolerance.

Genes involved in ergosterol and long-fatty acid metabolism and in cell wall biosynthesis and integrity were also identified as determinants of tolerance to formic acid. Changes in the saturation degree of fatty acid acyl chains, phospholipids and plasma membrane ergosterol content are believed to affect cell permeability to weak acids [15, 16, 59]. The increased saturation degree of fatty acids acyl chains leads to a more packed membrane structure that is more impermeable to weak acids [60]. Changes in the ergosterol content of plasma membrane influence its permeability and fluidity, and can even affect the activity of membrane transporters including those involved in multidrug resistance [59, 61].

Several genes involved in amino acid biosynthesis were also found to confer increased tolerance to formic acid, suggesting that this acid may cause a depletion of their pool, as reported for acetic acid [26, 62]. On the other hand, it is important to mention that weak acids inhibit amino acid uptake, by affecting the activity of the amino acids permeases, thus exacerbating the influence of the auxotrophic marks present in the parental strain in the susceptibility phenotypes [62, 63]. Since *S. cerevisiae* BY4741 and the derived deletion mutant collection have several auxotrophic markers, it is possible that the susceptibility phenotypes observed with strains where genes encoding proteins involved in amino acids homeostasis and synthesis are deleted are an artefact related with those auxotrophic markers.

Among the determinants of resistance identified in this chemogenomic analysis is the *VAM6* gene, encoding a guanine nucleotide exchange factor that is an activator of the Tor Complex 1 (TORC1) [64] and two genes encoding targets of TORC1 (*SCH9* and *RTG2*). The TORC1 pathway is activated in response to carbon and amino acid starvation and hyperosmotic and redox stresses (reviewed in [65]) and also regulates apoptosis in acetic acid-challenged cells [26]. Our results suggest that the TORC1 pathway may also mediate yeast response to formic acid, possibly in response to amino acid starvation or osmotic or oxidative stress-induced stimuli [21, 26].

The *HAA1* gene, encoding the main orchestrator of *S. cerevisiae* global response to acetic acid [15], was demonstrated to be a determinant of tolerance to formic acid, as well as the deletion of Haa1-target genes *TPO3*, *YGP1*, *SAP30* and *HRK1*. Moreover, the transcriptional levels from the above mentioned Haa1-target genes increase in response to formic acid and this transcriptional activation is dependent on Haa1, suggesting that the Haa1-regulon is also activated in response to formic acid stress.

A concentration of 30 mM of formic acid (at pH 4.0) was found to cause a latency period identical to the latency period induced by a higher concentration of acetic acid (60 mM) (see Additional file 5). Considering the p*K*_a_ (3.74) and LogP (−0.54) constants for formic acid (at pH 4.0), the higher toxicity of formic acid compared to acetic acid strongly suggests that there are mechanisms underlying formic acid toxicity that are specific to this weak acid. Among these hypothesized mechanisms specific to formic acid (C1) toxicity or tolerance that emerged from this work is a mechanism that has not been observed before for weak acids in general, not even for acetic acid (C2): the hypothesized uptake of formic acid through the high-affinity potassium transporter, Trk1. This unexpected proposed mechanism is supported by a number of evidences gathered during this study. Among them is the increased accumulation of radiolabeled formic acid in cells devoid of *TRK1*. The involvement of the Trk1 transporter in facilitating formic acid uptake is consistent with the role of genes encoding the positive (*SAT4*, *HAL3* and *HAL5*) and negative (*PPZ1*) regulators of Trk1 biological activity, in decreased or increased tolerance to formic acid, respectively. The lack of detectable effect of *TRK2* deletion in yeast tolerance to formic acid is also consistent with this hypothesized role of Trk1 as a facilitator of formic acid uptake into the cell given that Trk1 is a low-affinity K^+^ transporter, whereas Trk1 is a high-affinity K^+^ transporter. Remarkably, *S. cerevisiae* TRK-potassium transporters have been described as mediators of currents of different anions, including Cl^−^ ≫ formate > gluconate > acetate ≫ phosphate (order of selectivity for several slightly permeant anions at pH_0_ = 5.5) [66]. Our results also indicate that when the growth media has saturating concentrations of K^+^ (20 mM), the deletion of *TRK1* can be explored to increase yeast robustness against formic acid stress.

## Conclusion

This study provides the first genome-wide identification of determinants and mechanisms of formic acid toxicity and tolerance in yeast. Among the relevant insights obtained, the role of the Haa1 transcription factor and the Haa1-regulon in the adaptive response and tolerance to formic acid and the biological activity of Trk1 and of its positive regulators in increasing yeast susceptibility to formic acid are highlighted. Collectively, the indications obtained are considered useful to guide the design of fermentation media (increasing K^+^ concentration) and the genetic manipulation of yeast cell to obtain more robust strains (engineering the Haa1 regulon) for second-generation bio-ethanol production in lignocellulosic biorefineries. The deletion of the *TRK1* gene and of genes encoding Trk1 positive regulators is also suggested for maximal yeast tolerance to formic acid at concentrations of K^+^ for which ectopic uptake of K^+^ is sufficient to fulfil cellular requirements.

## Additional files

**Additional file 1: Table S1.**List of genes whose expression increases *S. cerevisiae* tolerance to formic acid based on the screening of the EUROSCARF deletion mutant collection (the elimination of the indicated genes increases yeast susceptibility to formic acid). The severity of growth inhibition is indicated by “+” or “++” and the level was attributed according to the criteria described in Additional file 3.

**Additional file 2: Table S2.** List of genes whose expression increases *S. cerevisiae* susceptibility to formic acid based on the screening of the EUROSCARF deletion mutant collection (the elimination of the indicated genes increases yeast tolerance to formic acid).

**Additional file 3: Table S3.** Primers used in this study.

**Additional file 4: Figure S1.** Description of the criteria used to define the different levels of susceptibility to formic acid of the deletion mutant strains. Wild-type and deletion mutant strains were spotted onto solid MMB medium (pH 4.5) supplemented with increasing concentrations of formic acid (60, 70 and 80 mM). Strains that did not grow in the presence of 70 mM of formic acid were considered susceptible strains (S); two levels of susceptibility where defined when mutant growth was reduced (+), or completely abolished (++), when cultivated in the presence of 60 mM of formic acid. Strains that grew in medium supplemented with 80 mM of formic acid were considered resistant strains (*R*).

**Additional file 5: Table S4.** Comparison of lag-phase duration of the parental strain BY4741 cells cultivated in the presence of equitoxic concentrations of short-chain monocarboxylic acids that cause a latency phase of approximately 17 h. The duration of the lag-phases was determined in cultures of non-adapted BY4741 cells cultivated in MM4 medium (pH 4.0) after sudden exposure to equitoxic concentrations of the listed weak acids (CT, in bold). [HA] concentration of the weak acid protonated at pH 4.0; LogP, logarithm of the partition coefficient of the weak acid between octanol and water.

## Abbreviations

ROS: Reactive oxygen species
FDH: Formate dehydrogenase
SGD: *Saccharomyces* genome database
KEGG: Kyoto encyclopedia for genes and genomes
GO: Gene ontology
MDR: MultiDrug resistance
MAP: Mitogen-activated protein
LogP: Coefficient of octanol–water partition
TORC1: Tor complex 1
