# Supplementary material for: Genome-wide search for candidate genes for yeast robustness improvement against formic acid reveals novel susceptibility (Trk1 and positive regulators) and resistance (Haa1-regulon) determinants
Source: Biotechnol Biofuels. 2017 Apr 19;10:96. doi: 10.1186/s13068-017-0781-5 (PMC5395885; doi:10.1186/s13068-017-0781-5)
Supplement: Supplementary file 3 — Additional file 3: Table S3. Primers used in this study. [file 13068_2017_781_MOESM3_ESM.docx]

**Table S3.** Primers used in this study.

| **Primer name** | **Sequence (5’-3’)** |
| --- | --- |
| **ACT1_fw** | CTCCACCACTGCTGAAAGAGAA |
| **ACT1_rv** | CCAAGGCGACGTAACATAGTTTT |
| **TPO2_fw** | TGAGTGATCAAGAATCTGTTG |
| **TPO2_rv** | CGGTACGGTTCAATTGCTTT |
| **TPO3_fw** | TTGTGACTGGCGATCCAGAA |
| **TPO3_rv** | ACTCCAACGGATCCATGCA |
| **COM2_fw** | TGTTTAACGAAAGCCGCAATAA |
| **COM2_rv** | GGCGTTGGCATTTGGTATG |
| **HRK1_fw** | TGGCCCATACCGTCTATTACG |
| **HRK1_rv** | TGCAGTATACGGGACATGATAGGT |
| **SAP30_fw** | TGATCTGCGCCCCAAGA |
| **SAP30_rv** | TCAGGAACGCATCCGTATACTCT |
| **SUR2_fw** | CAAGGCCCCTGCCATTAAT |
| **SUR2_rv** | CACTCATTTCCGGCAGCAA |
