# Supplementary figures and images for: Genome-wide search for candidate genes for yeast robustness improvement against formic acid reveals novel susceptibility (Trk1 and positive regulators) and resistance (Haa1-regulon) determinants
Source: Biotechnol Biofuels. 2017 Apr 19;10:96. doi: 10.1186/s13068-017-0781-5 (PMC5395885; doi:10.1186/s13068-017-0781-5)

## Slide 1
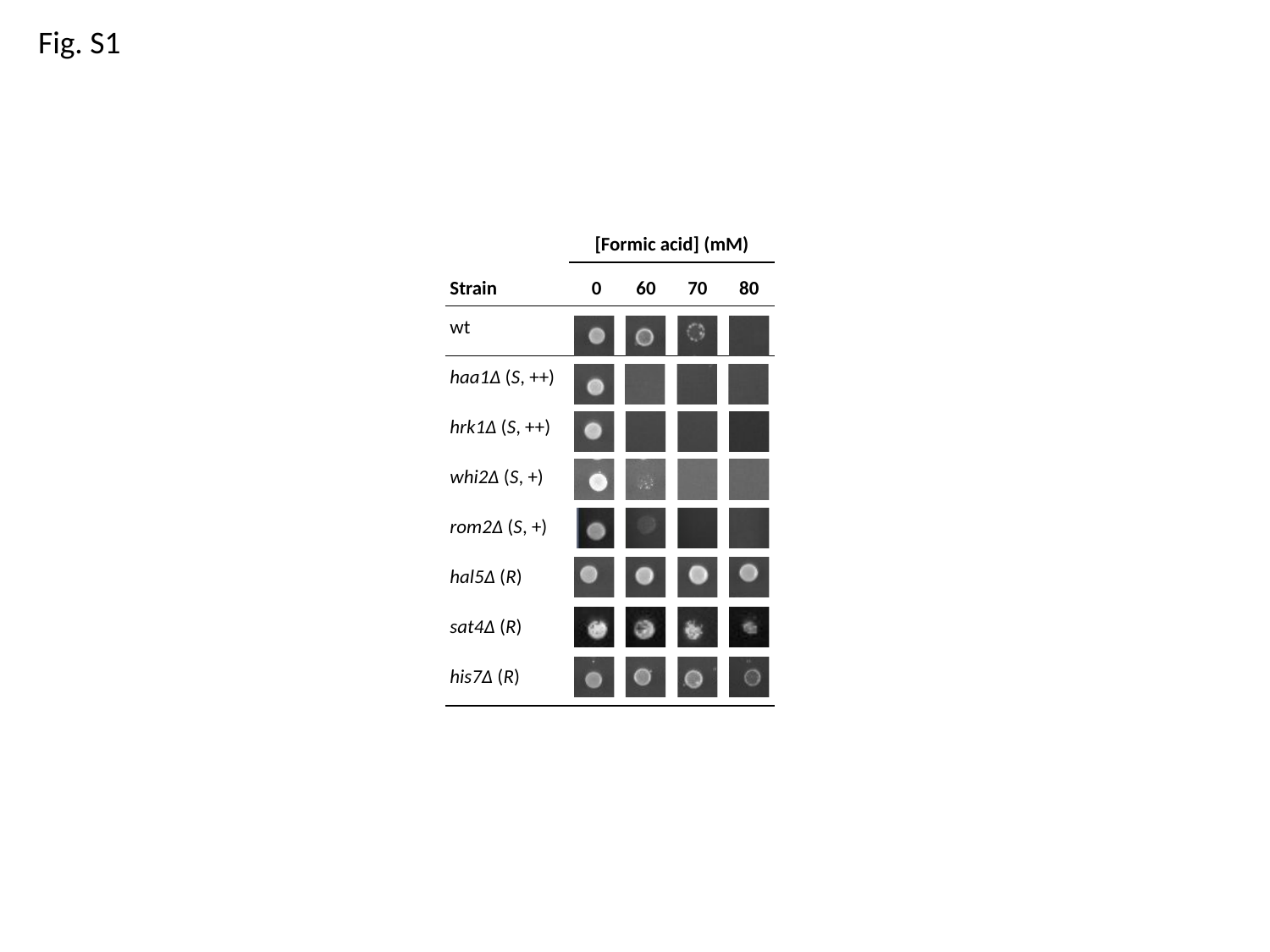

Fig. S1
| | [Formic acid] (mM) | | | |
| --- | --- | --- | --- | --- |
| Strain | 0 | 60 | 70 | 80 |
| wt | | | | |
| haa1Δ (S, ++) | | | | |
| hrk1Δ (S, ++) | | | | |
| whi2Δ (S, +) | | | | |
| rom2Δ (S, +) | | | | |
| hal5Δ (R) | | | | |
| sat4Δ (R) | | | | |
| his7Δ (R) | | | | |

Supplement: Supplementary file 4 — Additional file 4: Figure S1. Description of the criteria used to define the different levels of susceptibility to formic acid of the deletion mutant strains. Wild-type and deletion mutant strains were spotted onto solid MMB medium (pH 4.5) supplemented with increasing concentrations of formic acid (60, 70 and 80 mM). Strains that did not grow in the presence of 70 mM of formic acid were considered susceptible strains (S); two levels of susceptibility where defined when mutant growth was reduced (+), or completely abolished (++), when cultivated in the presence of 60 mM of formic acid. Strains that grew in medium supplemented with 80 mM of formic acid were considered resistant strains (R). [file 13068_2017_781_MOESM4_ESM.pptx]
