# Supplementary material for: Genome-wide search for candidate genes for yeast robustness improvement against formic acid reveals novel susceptibility (Trk1 and positive regulators) and resistance (Haa1-regulon) determinants
Source: Biotechnol Biofuels. 2017 Apr 19;10:96. doi: 10.1186/s13068-017-0781-5 (PMC5395885; doi:10.1186/s13068-017-0781-5)
Supplement: Supplementary file 5 — Additional file 5: Table S4. Comparison of lag-phase duration of the parental strain BY4741 cells cultivated in the presence of equitoxic concentrations of short-chain monocarboxylic acids that cause a latency phase of approximately 17 h. The duration of the lag-phases was determined in cultures of non-adapted BY4741 cells cultivated in MM4 medium (pH 4.0) after sudden exposure to equitoxic concentrations of the listed weak acids (CT, in bold). [HA] concentration of the weak acid protonated at pH 4.0; LogP, logarithm of the partition coefficient of the weak acid between octanol and water. [file 13068_2017_781_MOESM5_ESM.docx]

**Table S4.** **Comparison of lag-phase duration of the parental strain BY4741 cells cultivated in the presence of equitoxic concentrations of short-chain monocarboxylic acids that cause a latency phase of approximately 17h.** The duration of the lag-phases was determined in cultures of non-adapted BY4741 cells cultivated in MM4 medium (pH 4.0) after sudden exposure to equitoxic concentrations of the listed weak acids (C_T_, in bold). [HA] concentration of the weak acid protonated at pH 4.0; LogP, logarithm of the partition coefficient of the weak acid between octanol and water.

| Weak acid | Weak acid (mM) | | p*K*a  (20 °C) | LogP | Latency (h) BY4741 |
| --- | --- | --- | --- | --- | --- |
|  | **CT** | [HA]/CT (%) |  |  |  |
| Formic acid (C1) | **30** | 35 | 3.74^(b)^ | -0.54^(b)^ | 17^(d)^ |
| Acetic acid (C2) | **60** | 85 | 4.76^(a)^ | -0.24^(a)^ | 18^(c)^ |
| Propionic acid (C3) | **20** | 88 | 4.87^(a)^ | 0.32^(a)^ | 17^(c)^ |
| Butyric acid (C4) | **10** | 88 | 4.87^(a)^ | 0.83^(a)^ | 17^(c)^ |
| Octanoic acid (C8) | **0.55** | 89 | 4.88^(a)^ | 3.05^(a)^ | 17^(c)^ |

^(a)^ [70]; ^(b)^ [1]; ^(c)^ [27]; ^(d)^ (This study).
